# MiR‐106b inhibition suppresses inflammatory bone destruction of wear debris‐induced periprosthetic osteolysis in rats

**DOI:** 10.1111/jcmm.15376

**Published:** 2020-06-02

**Authors:** Binqing Yu, Jiaxiang Bai, Jian Shi, Jining Shen, Xiaobin Guo, Yu Liu, Gaoran Ge, Jiayi Lin, Yunxia Tao, Huilin Yang, Yaozeng Xu, Qiuxia Qu, Dechun Geng

**Affiliations:** ^1^ Department of Orthopedics The First Affiliated Hospital of Soochow University Suzhou China; ^2^ Department of Pharmacy The First Affiliated Hospital of Soochow University Suzhou China; ^3^ Jiangsu Institute of Clinical Immunology The First Affiliated Hospital of Soochow University Suzhou China

**Keywords:** bone formation, bone resorption, miR‐106b, periprosthetic osteolysis, wear debris

## Abstract

Aseptic loosening caused by periprosthetic osteolysis (PPO) is the main reason for the primary artificial joint replacement. Inhibition of inflammatory osteolysis has become the main target of drug therapy for prosthesis loosening. MiR‐106b is a newly discovered miRNA that plays an important role in tumour biology, inflammation and the regulation of bone mass. In this study, we analysed the in vivo effect of miR‐106b on wear debris‐induced PPO. A rat implant loosening model was established. The rats were then administrated a lentivirus‐mediated miR‐106b inhibitor, miR‐106b mimics or an equivalent volume of PBS by tail vein injection. The expression levels of miR‐106b were analysed by real‐time PCR. Morphological changes in the distal femurs were assessed via micro‐CT and histopathological analysis, and cytokine expression levels were examined via immunohistochemical staining and ELISA. The results showed that treatment with the miR‐106b inhibitor markedly suppressed the expression of miR‐106b in distal femur and alleviated titanium particle‐induced osteolysis and bone loss. Moreover, the miR‐106b inhibitor decreased TRAP‐positive cell numbers and suppressed osteoclast formation, in addition to promoting the activity of osteoblasts and increasing bone formation. MiR‐106b inhibition also significantly regulated macrophage polarization and decreased the inflammatory response as compared to the control group. Furthermore, miR‐106b inhibition blocked the activation of the PTEN/PI3K/AKT and NF‐κB signalling pathways. Our findings indicated that miR‐106b inhibition suppresses wear particles‐induced osteolysis and bone destruction and thus may serve as a potential therapy for PPO and aseptic loosening.

## 1 INTRODUCTION

Currently, artificial joint replacement (AJR) is considered the most effective surgical process to treat end‐stage osteoarthritis.^1^, ^2^ However, as the number of patients undergoing AJR increases, so does the number of patients requiring revision due to failed artificial joints.^3^ Previous studies have shown that aseptic loosening caused by periprosthetic osteolysis (PPO) is the main reason for shortening the service time of the joint prosthesis and revision of the primary AJR.^4^ Chronic friction in the artificial joint leads to erosion of the material, as well as the generation of tiny metal or polyethylene wear particles. These particles accumulate around the prosthesis and cause chronic macrophage‐mediated inflammation, which enhances the activation of osteoclasts and down‐regulates the function of osteoblasts, eventually leading to the occurrence of PPO and loosening of the prosthesis.^5^, ^6^, ^7^ Since wear of prosthesis is often unavoidable, inhibition of inflammatory osteolysis has become the main target of drug therapy for prosthesis loosening.^8^

MicroRNAs (miRNAs) are single‐stranded small RNA molecules with a length of about 22 nucleotides. They are categorized as non‐coding RNA and are currently the most studied non‐coding RNAs. The combination of miRNA and RNA represses the mRNA translation of genes and ultimately inhibits the protein expression of target genes. This allows the miRNAs to participate in the regulation of a variety of life processes, including inflammatory responses, osteocyte differentiation and occurrence of various diseases.^9^, ^10^, ^11^, ^12^ MiR‐106b is a newly discovered miRNA that is a member of the miR‐106b‐25 cluster and is significantly elevated in lung, stomach, liver and kidney cancers.^13^, ^14^ Recently, studies have demonstrated that miR‐106b plays an important role in tumour biology, inflammation and the regulation of bone mass.^15^, ^16^, ^17^ In addition, we have shown that miR‐106b partially suppresses bone formation in osteoporosis mice,^18^ and inhibition of miR‐106b expression reduces joint inflammation and bone destruction in collagen‐induced arthritis (CIA) mice.^19^ However, the role miR‐106b plays in PPO is not fully understood. This study aimed to clarify the role of miR‐106b in wear debris‐induced PPO, as well as the underlying mechanism by which miR‐106b regulates the inflammatory response and the balance between osteoblasts and osteoclasts.

## 2 MATERIALS AND METHODS

### 2.1 Rat implant wear debris model and drug treatments

A rat implant wear debris model was established as described previously.^20^ Forty‐eight male Sprague Dawley rats (400‐425 g) were implanted with titanium (Ti) rods (length 15 mm, diameter 1.5 mm; Goodfellow Company, USA) in the bilateral femoral bone. The proximal 10 mm of the Ti rod was pressed into the marrow cavity of the femoral bone to ensure a firm fixation. All of the rats were randomized into the following groups: control group (no Ti particles), vehicle group (with Ti particles), miR‐106b mimics‐treated group (with Ti particles) and miR‐106b inhibitor‐treated group (with Ti particles). The last three groups received an intramedullary injection of Ti particles (90% <30 μm; Alfa Aesar). Lentivirus‐mediated miR‐106b inhibitor (1 × 10^8^ U/mL), miR‐106b mimics (1 × 10^8^ U/mL) or an equivalent volume of PBS (0.2 mL) was administered to rats via tail vein injection in the first three days of the first and fourth week. The rats were euthanized six weeks after surgery, and the distal femur was retained for further study.^21^ All animals were provided by the Laboratory Animal Research Center of Soochow University (Suzhou, China). All animal experiments were approved by the Ethics Committee of the First Affiliated Hospital of Soochow University.

### 2.2 Real‐time PCR

The distal femur tissues were collected from six rats in each group and cryopreserved in liquid nitrogen. After weighing, the distal femurs were ground into powder in a mortar with added liquid nitrogen. Then, we added 8 mL TRIzol (Sigma) after the liquid nitrogen volatilizes to extract total RNA. A miRNA Isolation kit (Ambion) was used to isolate total miRNA according to the manufacturer recommendation. Quantitative RT‐PCR analysis for miRNAs was performed using a TP800 PCR Thermal Cycler Dice Detection System (Takara) and SYBR RT‐PCR kits (Takara). Stem‐loop primers for miR‐106b and U6 were brought from Ambion.

### 2.3 Determination of serum index

Before euthanasia, peripheral blood was obtained from each animal. The serum levels of tumour necrosis factor alpha (TNF‐α), interleukin (IL)‐1β and IL‐6 were analysed by flow cytometry. The serum levels of receptor activator of nuclear factor‐κ B ligand (RANKL), osteoprotegerin (OPG), osteocalcin (OCN), N‐terminal propeptide of type I procollagen (P1NP) and c‐terminal telopeptide of type I collagen (CTX), as well as the activity of TRAP5b, were assessed using specific ELISA kits (R&D Systems), according to the manufacturers’ instructions.

### 2.4 Liver and kidney toxicity

Peripheral blood was obtained, and routine blood biochemical tests were performed to assess hepatorenal toxicity. The white blood cell count, haemoglobin, platelet count, and levels of aspartate aminotransferase, alanine aminotransferase, blood urea nitrogen and creatinine were all measured.

### 2.5 Micro‐CT scanning

The distal femur specimens (n = 12 per group, six from right femur and six from left femur) were evaluated using a SkyScan1176 micro‐CT (SkyScan). The specimens were fixed with 10% formaldehyde for 24 hours and then scanned at an equidistant resolution of 9 μm. The X‐ray was operated at 80 kV and 100 μA. The three‐dimensional images were reconstructed using the CT analyzer software (SkyScan). The bone mineral density (BMD) and morphology in the volume of interest, as well as bone volume/total volume (BV/TV), trabecular thickness (Tb.Th) and bone surface/bone volume (BS/BV), were determined in order to evaluate the bone quality.

### 2.6 Histology and immunochemical analysis

The distal femur was examined by histological and immunochemical analyses after complete micro‐CT scanning. The left femurs of the rats were embedded in resin for hard tissue sectioning (n = 6 per group). Then the specimen was cut using a hard tissue slicer (EXAKT 300CP, Exakt‐Apparatebau Hermann) into 10‐μm‐thick sections along the vertical axis. After staining with toluidine blue, the sections were imaged using a Zeiss microscope (AXIOVERT 40C; Zeiss). The bone‐implant contact (BIC) and the percentage of new bone area were quantified using image analysis software Image Pro‐Plus 6.0 (Media Cybernetics).

The distal femur (right femurs of the rats, n = 6 per group) was fixed in 10% formaldehyde for 24 hours, decalcified in 10% EDTA for three weeks and then embedded in paraffin. Tissue sections (5 mm) were cutted using a microtome (LEICA RM2165, LEICA, Nussloch, Germany) and analysed histologically by various staining methods, including haematoxylin and eosin (H&E), tartrate‐resistant acid phosphatase (TRAP), immunohistochemistry and immunohistofluorescence, as previously described.^22^ The stained sections were observed and photographed using a Zeiss AXIOVERT 40C microscope. Image Pro‐Plus 6.0 was used to measure trabecular number (Tb.N), trabecular separation (Tb.Sp), erosion surface/bone surface (ES/BS), the ratio of osteoclast number to bone surface (OCs/BS, mm^‐2^) and the ratio of osteoclast area to bone surface (OCs/BS, %), following the methods described previously.^23^

### 2.7 Immunohistochemical staining

Tissue sections were treated with 5% hydrogen peroxidase at 37°C for 10 minutes for antigen restoration. The tissue sections were then incubated with primary antibodies against cathepsin K (CTSK, 1:500), matrix metallopeptidase 9 (MMP‐9, 1:500), alkaline phosphatase (ALP, 1:500), osteocalcin (OCN, 1:100), RANKL (1:500), osteoprotegerin (OPG, 1:500), TNF‐α (1:500), IL‐1β (1:500), IL‐6 (1:600), PTEN (1:100), p‐AKT (1:250), p‐IκB‐α (1:100) or p‐p65 (1:1000) for 12 hours at 4°C. The sections were washed with PBS the next day and then incubated with the appropriate secondary antibody for 30 minutes at room temperature, after which haematoxylin was used for the counterstain. All antibodies were purchased from Abcam. Four consecutive slices were scored semi‐quantitatively for each antibody by two pathologists. A score of 0 represented the minimal expression, 1 represented the mild expression, 2 represented the moderated expression, and 3 represented the abundant expression.^24^

### 2.8 Immunohistofluorescence staining

After antigen retrieval (as described above), tissue sections were incubated with antibodies against anti‐F4/80 (1:50) and either anti‐iNOS (1:100) or anti‐CD206 (1:1000) overnight at 4°C. The sections were then incubated for 30 minutes with the respective goat anti‐rat IgG H&L (Alexa Fluor 488; Abcam) or goat anti‐rabbit IgG H&L (Alexa Fluor 647, Abcam) at 37°C and then counterstained with 4’,6‐diamidino‐2‐phenylindole to mark the nucleus. Confocal microscopic images were collected with a Zeiss laser scanning microscope (LSM 510; Zeiss), and the images were analysed using the LSM 5 Release 4.2 software.

### 2.9 Statistical analysis

SPSS 22.0 (SPSS) was used for data analysis. All of the data are expressed as the mean ± standard deviation (SD). Statistical analyses were performed using two‐tailed Student's t tests or one‐way analysis of variance (ANOVA) followed by Tukey's post hoc test. A *P* value < 0.05 was considered to be significant.

## 3 RESULTS

### 3.1 MiR‐106b inhibition alleviated Ti particle‐induced osteolysis and bone loss in vivo

**FIGURE 1:**
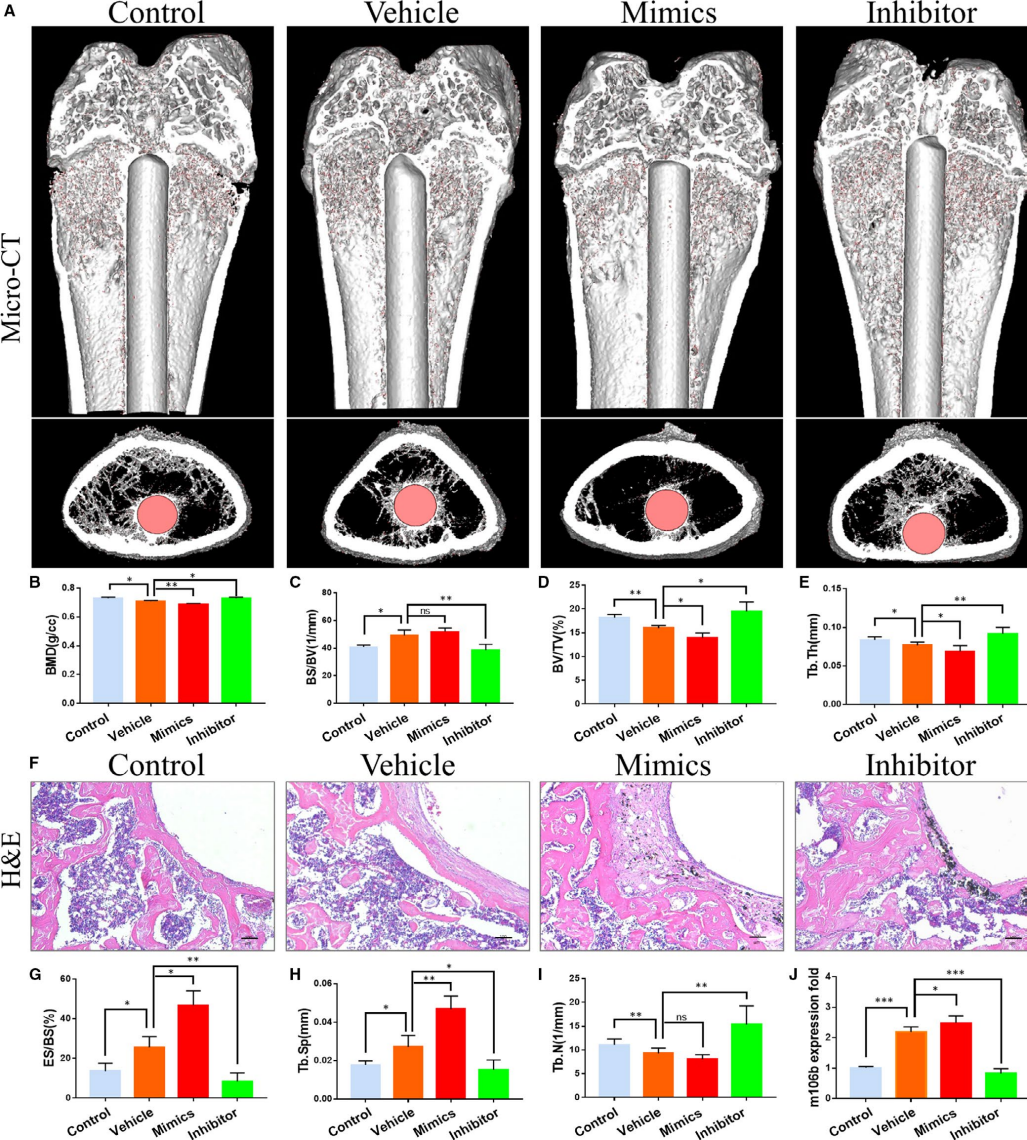
MiR‐106b inhibition alleviated Ti particle‐induced osteolysis and bone loss in vivo. A, Representative 3D reconstruction of micro‐CT images. B, BMD. C, BS/BV. D, BV/TV and E, Tb.Th within ROI were calculated, n = 12 per group. F, Representative slices with H&E staining. Scar bar: 100 μm. G, ES/BS. H, Tb.Sp and I, Tb.N were calculated. n = 6 per group. J, Quantitative real‐time PCR analysis of miR‐106b expression in distal femur tissues from rats in different groups, n = 6 per group. *P < .05, **P < .01, ***P < .001

Micro‐CT reconstructions of rat femurs showed aggravation of the structural damages of the trabecular bone around Ti rods, and the erosion range was significantly increased after the Ti particles implantation (Figure 1A). After treatment with the miR‐106b inhibitor, trabecular bone loss around the Ti rods was significantly diminished, suggesting that the miR‐106b inhibitor was able to reduce osteolysis caused by the Ti particles. Statistical analysis showed that the BMD, trabecular thickness (Tb.Th) and BV/TV of the vehicle group implanted Ti particles were significantly lower than those of the control group. However, after treatment with miR‐106b inhibitor, osteolysis was decreased and the BMD (0.7301 ± 0.0088 g/cc vs 0.7078 ± 0.0061 g/cc for miR‐106b inhibitor group vs vehicle group, similarly hereinafter), BS/BV (38.57 ± 4.41% vs 49.43 ± 3.61%), BV/TV (19.46 ± 1.975% vs 15.95 ± 0.33%) and Tb.Th (0.09161 ± 0.00510 mm vs 0.07672 ± 0.00246 mm) were dramatically reversed from those in the vehicle group (*P* < .05). As expected, the trends were opposite in the miR‐106b mimics group, suggesting that miR‐106b exacerbated osteolysis around the Ti rods (Figure 1B‐E).

Histological staining showed inflammation around the Ti particles implanted area (which was expected), with a large fibrous capsule between the bone structure and Ti rod and alteration of bone continuity. In contrast, the miR‐106b inhibitor‐treated group exhibited a reduction in the inflammatory response and less bone destruction (Figure 1F). Histomorphometric analysis showed that the ES/BS was 25.56 ± 3.30% in the vehicle group, 13.64 ± 2.06% in the control group, 8.41 ± 3.05% in the miR‐106b inhibitor group and 46.76 ± 4.28% in the miR‐106b mimics group, being all the calculated deviations statistically significant (Figure 1G). The measurement results also showed that the miR‐106b inhibitor‐treated group had the lowest Tb.Sp (0.01531 ± 0.00291 μm) and the highest Tb.N (15.35 ± 2.28 mm^‐1^), and the difference was statistically significant compared with the vehicle group (*P* < .05) (Figure 1H,I).

To verify whether miR‐106b signalling pathway is involved in osteolysis induced by titanium particles in *vivo*, we detected the expression of miR‐106b in distal femur, and found higher expression levels in vehicle group compared with control group. Then, we tried to regulate the production of endogenous miR‐106b by injecting miR‐106b inhibitor or mimics. As expected, the endogenous miR‐106b was significantly decreased in miR‐106b inhibitor group and significantly increased in miR‐106b mimics group compared with vehicle group (Figure 1J).

### 3.2 MiR‐106b inhibition decreased osteoclast numbers and suppressed osteoclast formation

**FIGURE 2:**
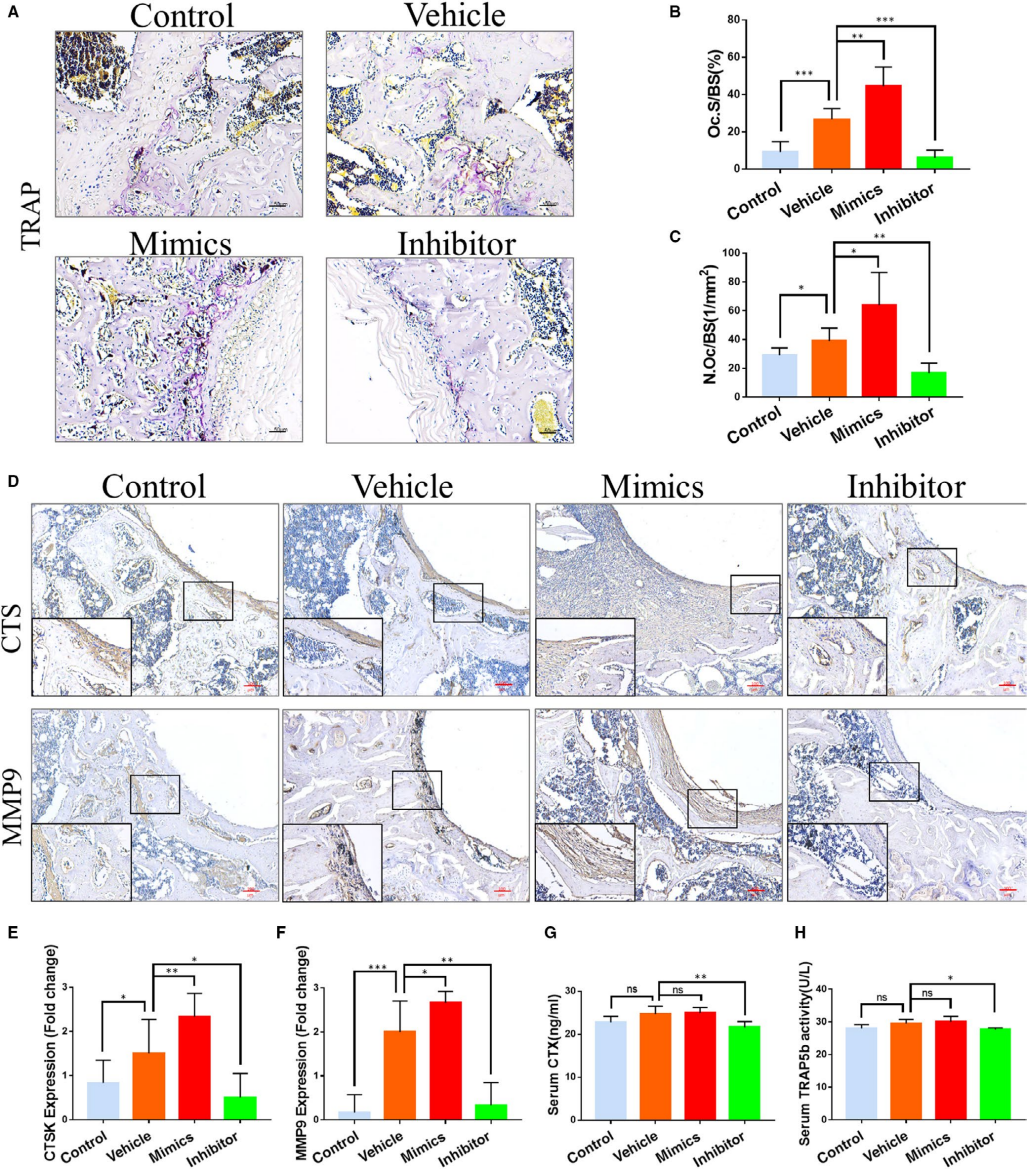
MiR‐106b inhibition decreased osteoclast numbers and suppressed osteoclast formation. A, Representative slices with TRAP staining. Scale bar: 50 μm. B, Oc.S/BS and C, N.Oc/BS were calculated. D, Representative immunohistochemical images of CTSK and MMP‐9. Scar bar: 100 μm. E,F, Semi‐quantitative analysis of CTSK and MMP‐9 expression. G, Serum levels of CTX and H, TRAP5b in each group were determined. n = 6 per group. *P < .05, **P < .01, ***P < .001

Osteoclasts are the direct effector cells mediating wear particles‐induced osteolysis.^25^ Thus, we examined the ability of miR‐106b to regulate osteoclast activity in vivo. Histological results showed large continuous TRAP‐positive areas around the Ti implants were observed in the vehicle and miR‐106b mimics‐treated groups and only a few TRAP‐positive cells were found in the miR‐106b inhibitor‐treated and control group (Figure 2A). Bone tissue quantitative analysis showed that Oc.S/BS and N.Oc/BS remarkably increased after the addition of Ti particles and significantly decreased after treatment with the miR‐106b inhibitor (26.67 ± 3.37% vs 6.18 ± 2.47%; 39.06 ± 5.15 mm^‐2^ vs 16.84 ± 4.07 mm^‐2^, *P* < .05) (Figure 2B,C). In addition, immunohistochemical staining results showed that CTSK and MMP‐9 expression around the Ti implants were also significantly inhibited after treatment with the miR‐106b inhibitor (Figure 2D–F). Corroborating the above mentioned results, serum levels of CTX‐1 and TRAP5b, well known as bone resorption markers, decreased significantly after treatment with the miR‐106b inhibitor (Figure 2G,H). Our results indicated that the miR‐106b inhibitor lightened osteolysis caused by Ti particles by inhibiting the formation of osteoclasts in vivo.

### 3.3 Effects of miR‐106b on osteogenic activity

**FIGURE 3:**
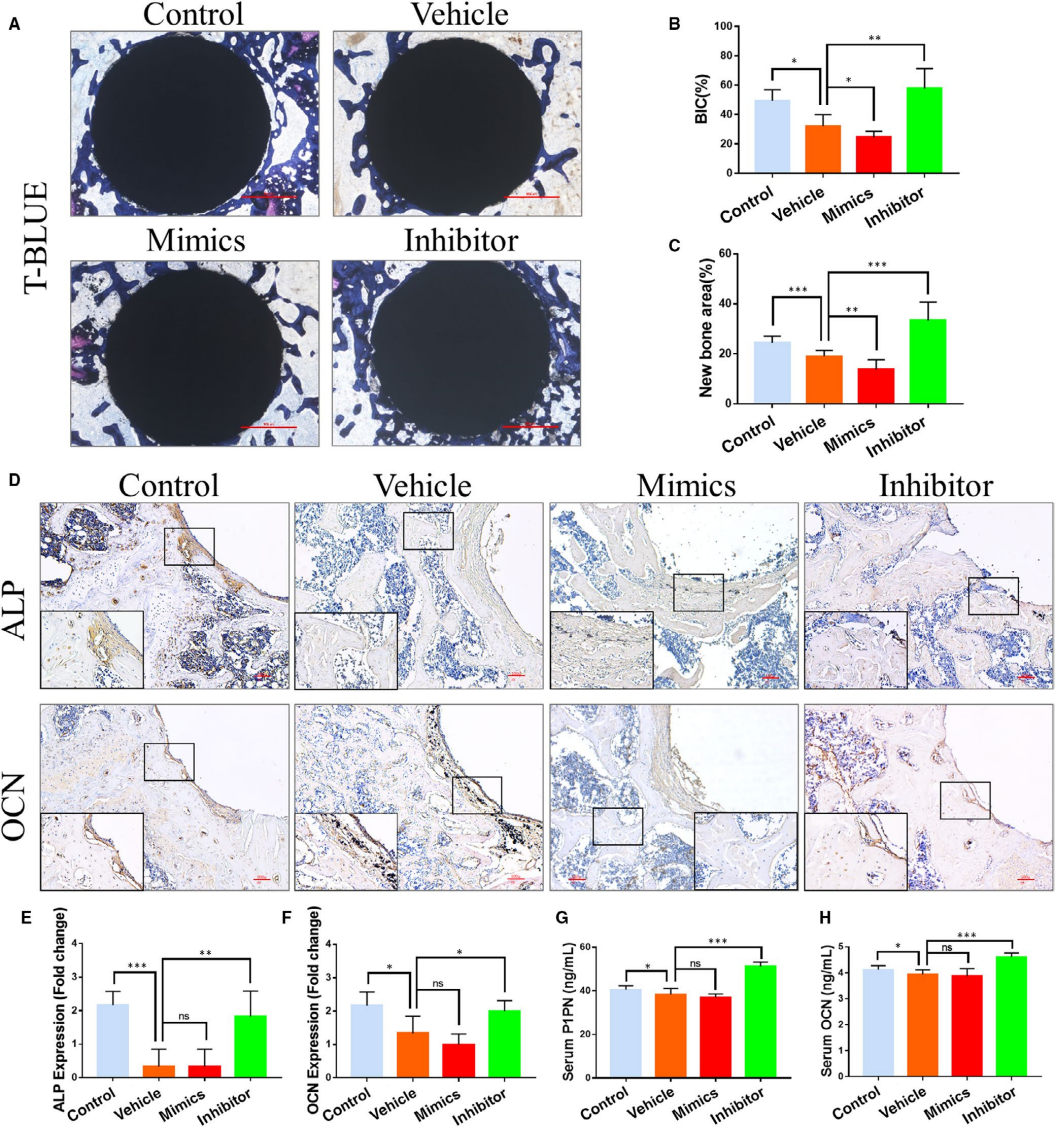
Effects of miR‐106b on osteogenic ability in vivo. A, Representative histological images of Ti rods with toluidine blue stain. Scar bar: 500 μm. B, Average histomorphometric values of BIC and C, quantitative analysis of the new bone area were determined. D, Representative immunohistochemical images of ALP and OCN. Scar bar: 100 μm. E, F, Semi‐quantitative analysis of ALP and OCN expression. G, H, Serum levels of OCN and P1NP in each group. n = 6 per group. *P < .05, **P < .01, ***P < .001

Since a decrease in new bone formation is one of the main pathological changes of wear particles‐induced osteolysis,^26^ we determined whether inhibition of miR‐106b has the ability to promote osteogenesis around the loosening implant. Toluidine blue staining revealed that there was a reduction in the bone matrix around the implant in the vehicle and the miR‐106b mimics‐treated group. While, more trabecular bone was connected on the implant surface in the miR‐106b inhibitor‐treated group (Figure 3A). Quantitative analysis showed that the miR‐106b inhibitor‐treated group had almost twice the bone‐implant contact (BIC) as the vehicle group (Figure 3B). In addition, there was also a significant increase in the percentage of the new bone area after treatment with the miR‐106b inhibitor (Figure 3C).

Immunohistochemical results showed that expression of ALP and Osterix (an osteoblast‐specific transcription factor) was almost completely absent around the Ti implants in the vehicle‐ and mimics‐treated groups. Interestingly, the number of ALP‐ and Osterix‐positive cells in the osteolysis area surrounding the femoral implant increased significantly, which is good because miR‐106b inhibitor treatment (Figure 3D–F). We further tested the serum levels of protein OCN and peptide P1NP and observed that Ti particle implantation caused a significant decrease in the levels of OCN and P1NP. After treatment with the miR‐106b inhibitor, the levels of P1NP and OCN increased by 18.4% and 35.3% compared with control or vehicle, respectively (Figure 3G,H). Taken together, these results indicated that miR‐106b inhibition promoted the osteogenic ability of osteoblasts, as well as bone formation around the Ti implants.

### 3.4 MiR‐106b altered the RANKL/OPG balance

**FIGURE 4:**
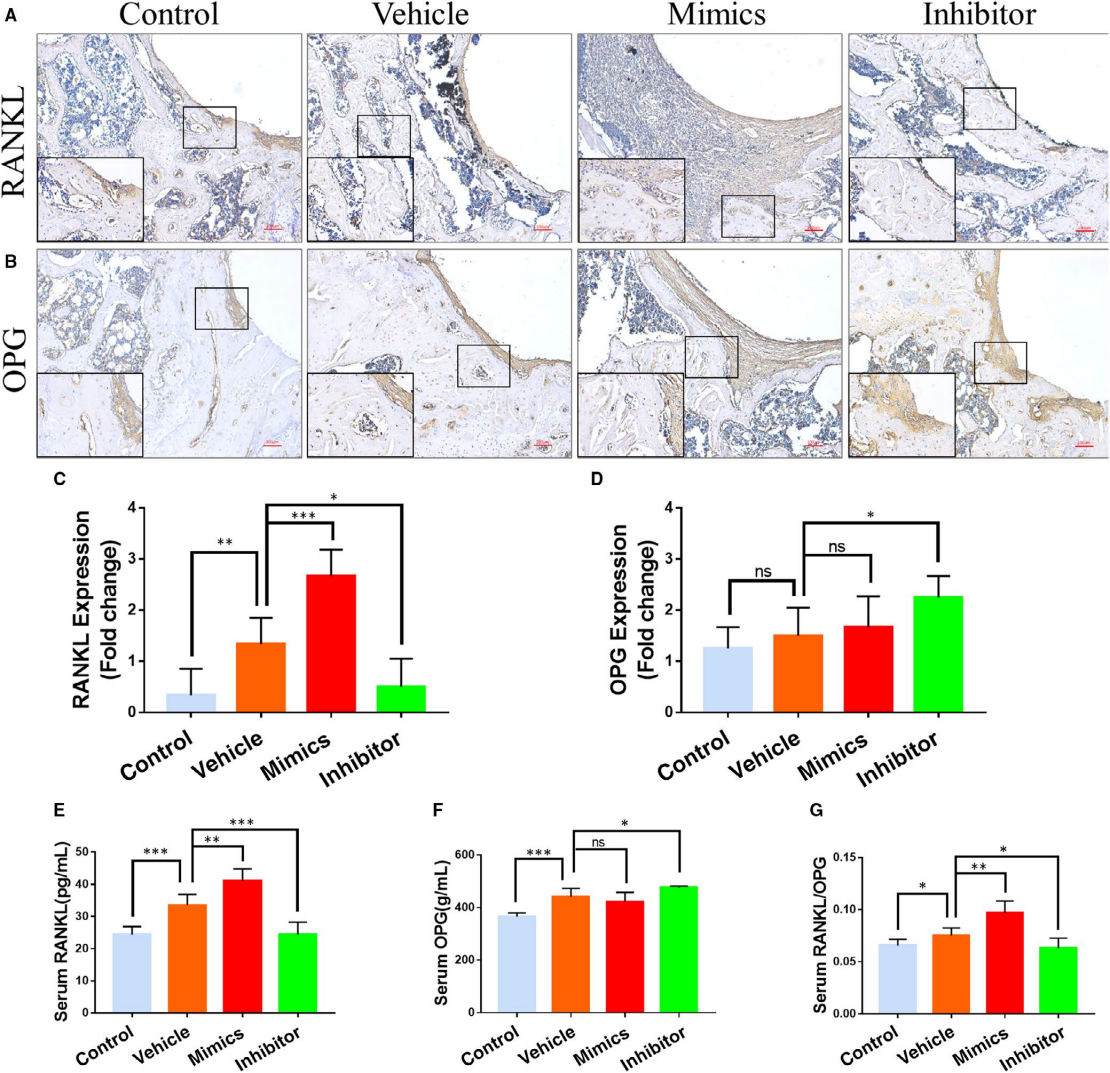
MiR‐106b affected RANKL/OPG balance in vivo. A, B, Representative immunohistochemical images of RANKL and OPG. Scar bar: 100 μm. C, D, Semi‐quantitative analysis of RANKL and OPG expression. E‐G, Serum levels of RANKL and OPG and the RANKL:OPG ratio in each group. n = 6 per group. *P < .05, **P < .01, ***P < .001

RANKL/OPG is a key signalling pathway regulating wear particles‐induced osteolysis.^27^ Immunohistochemical staining showed that after the addition of Ti particles, an extensive RANKL expression was observed (Figure 4A,C), with a slight up‐regulated expression of OPG (Figure 4B,D). In contrast, the miR‐106b inhibitor treatment group exhibited a significant decrease in the numbers of RANKL‐positive cells but a marked increase in OPG‐positive cells when compared with the vehicle group. ELISA results showed that in the peripheral blood from the vehicle group both RANKL and OPG expression was significantly increased compared with the control group. Furthermore, the RANKL/OPG ratio in the vehicle group increased compared with the control group. However, after treating rats with the miR‐106b inhibitor, the OPG level increased by 9%, while the RANKL level reduced by 27.2%. Therefore, the RANKL/OPG ratio of the Ti particle‐stimulated group decreased from 0.0745 ± 0.0079 to 0.0626 ± 0.0128 (Figure 4E‐G). In contrast, the RANKL/OPG ratio was 0.0962 ± 0.0126 in the miR‐106b mimics‐treated group, which was significantly higher than that in the vehicle and miR‐106b inhibitor‐treated groups.

### 3.5 MiR‐106b inhibition regulated macrophage polarization and decreased the inflammatory response

**FIGURE 5:**
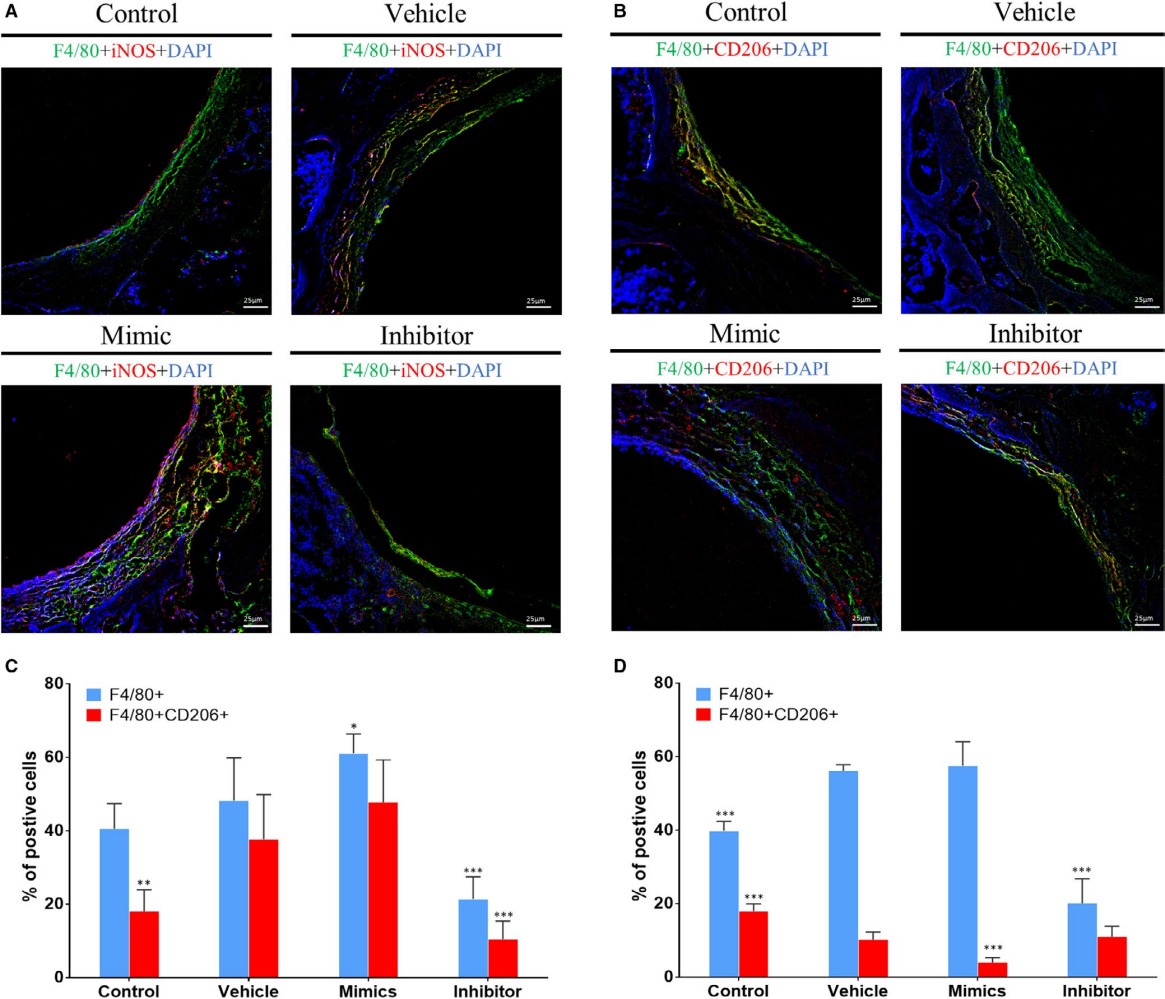
MiR‐106b inhibition regulated macrophages repolarization. A, B, Representative immunohistofluorescence stain images of F4/80, iNOS and CD206. Scar bar: 25 μm. C, D, The percentage of F4/80+/iNOS + M1 macrophage and F4/80+/CD206 + M2 macrophages was counted. n = 6 per group. *P < .05, **P < .01, ***P < .001; compared with the vehicle group

According to the above results, miRNAs can regulate both the activity of osteoclasts and osteoblasts. Interestingly, inflammation plays a key role in the balance of osteoblasts and osteoclasts, especially in the process of PPO.^28^, ^29^ Thus, we have been suggested that miR‐106b acts to suppress osteoclast formation and promote new bone formation by regulating inflammatory cytokine expression. Macrophages are a group of immune cells that can polarize into different phenotypes under various microenvironments and pathological conditions and, playing, then, an important role in inflammatory bone destruction.^30^ The classical M1 phenotype is pro‐inflammatory, while the M2 phenotype is anti‐inflammatory. In order to verify whether inhibition of miR‐106b has an effect on macrophage polarization during inflammatory osteolysis, antibodies against F4/80 and either iNOS or CD206 were used for immunohistofluorescent staining. As shown in Figure 5A,B, F4/80 marked a substantial number of macrophages in the fibrous capsule around the Ti implants of vehicle and miR‐106b mimics‐treated groups. The majority of these stained macrophages were the iNOS‐positive M1 phenotype, and only a few expressed the CD206‐positive M2 phenotype. On the other hand, after miR‐106b inhibitor treatment, the expression of F4/80 slightly decreased; however, the proportion of CD206 + M2 macrophages increased, and the proportion of iNOS + M1 macrophages decreased (Figure 5C,D).

**FIGURE 6:**
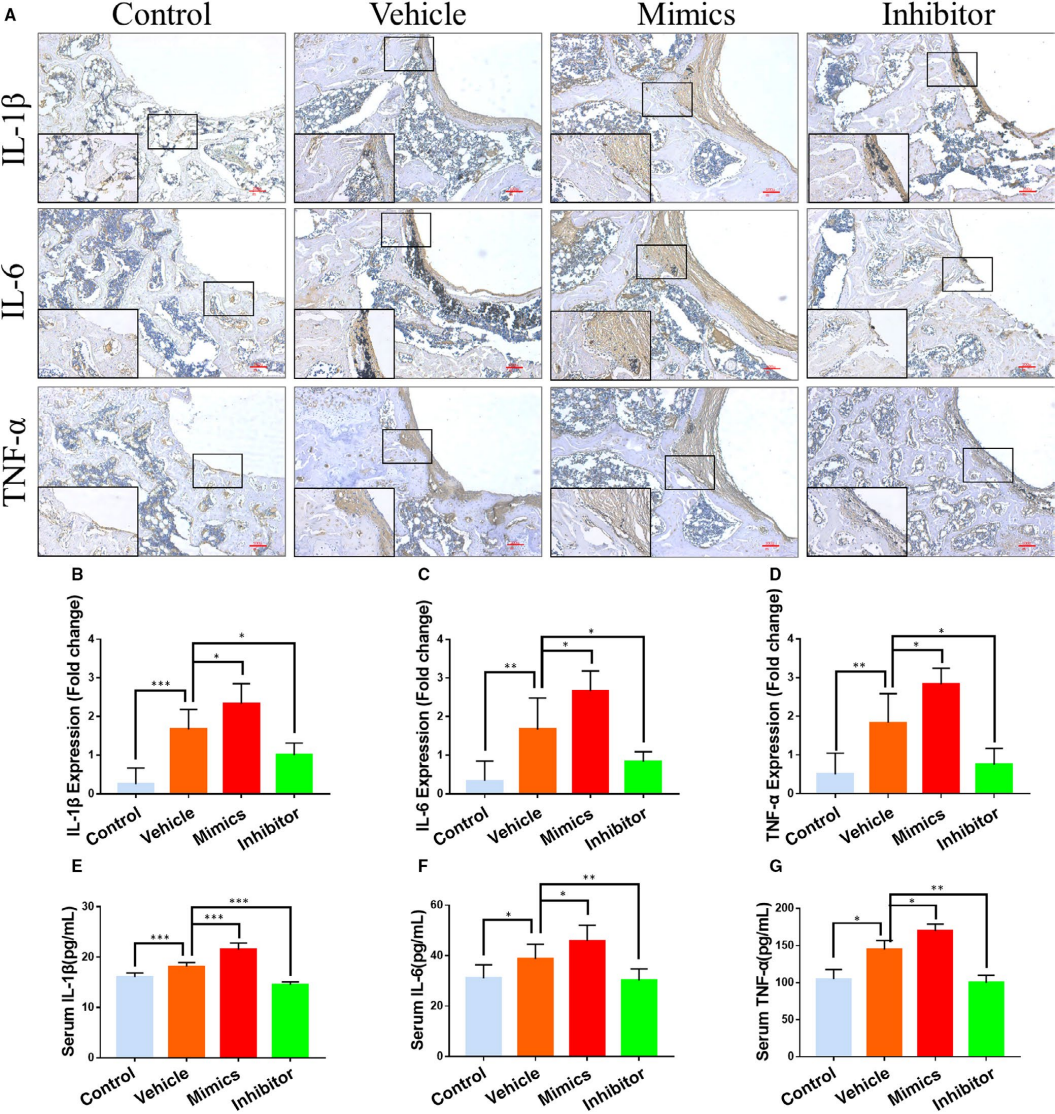
MiR‐106b inhibition suppressed inflammatory cytokines expression in vivo. (A) Representative immunohistochemical images of TNF‐α, IL‐1β and IL‐6. Scar bar: 100 μm. B‐D, Semi‐quantitative analysis of TNF‐α, IL‐1β and IL‐6 expression. E‐G, Serum levels of TNF‐α, IL‐1β and IL‐6 in each group. n = 6 per group. *P < .05, **P < .01, ***P < .001

As expected, it was found that the expression of inflammatory factors (TNF‐α, IL‐1β and IL‐6) was significantly increased in the fibrous capsule and the bone matrix of vehicle and miR‐106b mimics‐treated groups; however, minimal positive staining reactions of those factors were observed in the miR‐106b inhibitor‐treated group. In contrast, treatment with miR‐106b mimics increased the positive staining of TNF‐α, IL‐1β and IL‐6 in the fibrous capsule (Figure 6A‐D). The expression of inflammatory factors in the serum was also significantly reduced after treating rats with the miR‐106b inhibitor, indicating that miR‐106b inhibition promoted the polarization of macrophages from M1 to M2, thereby decreasing the inflammatory response in vivo (Figure 6E‐G).

### 3.6 Activation of PTEN/PI3K/AKT and NF‐κB signalling pathways

**FIGURE 7:**
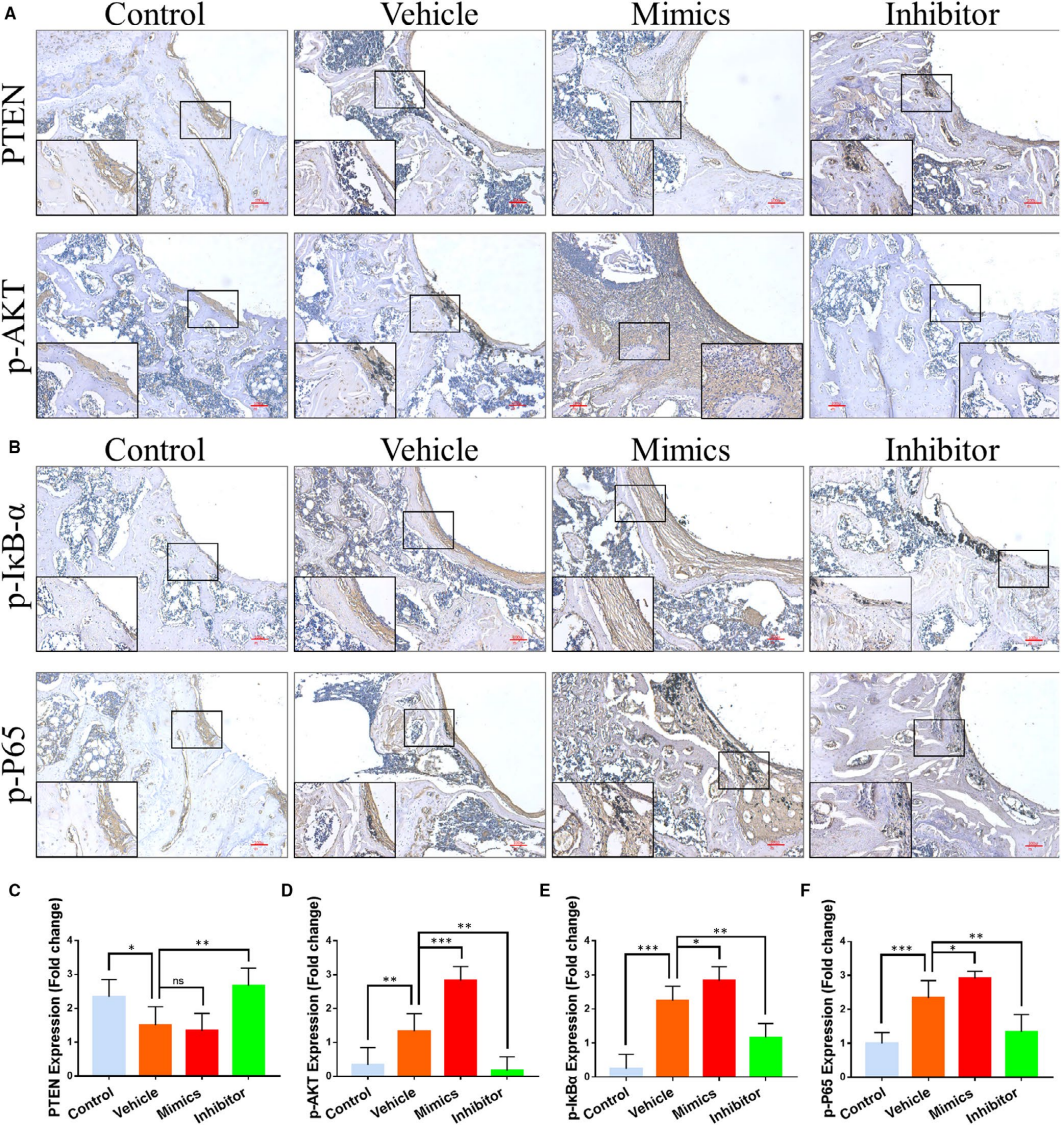
MiR‐106b and the activation of PTEN/PI3K/AKT and NF‐κB signalling pathways in vivo. A, Representative immunohistochemical images of PTEN and p‐Akt. Scar bar: 100 μm. B, Representative immunohistochemical images of p‐IκBα and p‐P65. Scar bar: 100 μm. C‐F, Semi‐quantitative analysis of PTEN, p‐Akt, p‐IκBα and p‐P65 expression. n = 6 per group. *P < .05, **P < .01, ***P < .001

PTEN is one of the negative regulatory factors of the PI3K/Akt pathway. Previous study has already confirmed that miR‐106b has a negative regulatory effect on PTEN.^31^ Thus, we aimed to determine whether the inhibition of bone destruction was achieved through the regulation of the PTEN/PI3K/AKT signalling pathway.^32^ In the implant wear debris model, we observed only a small area of intensely stained PTEN areas dispersed around the implanted materials, and the expression of PTEN was significantly up‐regulated in the miR‐106b inhibitor group (Figure 7A,C). In addition, there was a significant staining around the implanted materials, indicating a strong immune response involving p‐Akt in the vehicle and miR‐106b mimics‐treated groups; however, few positive staining reactions were observed in miR‐106b inhibitor‐treated group (Figure 7A,D). In summary, these results suggested that inhibition of miR‐106b activated PTEN, which, in turn, blocked the Akt signalling pathway in vivo.

It has been shown that the PI3K/AKT signalling pathway mediates the activation of NF‐κB,^33^ an important signalling pathway regulating inflammatory bone destruction.^34^, ^35^, ^36^, ^37^ To explore whether the suppression of macrophage polarization caused by the miR‐106b inhibitor was induced by the NF‐κB signalling pathway, we tested the phosphorylation status of IκB‐α and p65—two molecules that play a pivotal role in the NF‐κB signalling pathway.^38^ Immunohistochemistry staining demonstrated that the miR‐106b inhibitor significantly suppressed the positive staining of p‐IκB‐α and p‐p65 around the Ti implants (Figure 7B,E,F). Thus, our experimental results strongly suggest that miR‐106b inhibition suppressed inflammatory bone destruction through both the PTEN/PI3K/Akt and NF‐κB signalling pathways.

### 3.7 Liver and kidney toxicity in rats

The results of the routine blood analysis showed that the changes of white blood cell count, haemoglobin and platelet count were not statistically significant (*P* > .05) after treatment with either the miR‐106b inhibitor or miR‐106b mimics. There were also no significant changes in the levels of aspartate aminotransferase, alanine aminotransferase, blood urea nitrogen and creatinine, which indicated that the miR‐106b inhibitor and miR‐106b mimics had no effect on the liver and kidney function in rats (Table 1).

**TABLE 1:** Toxicity of miR‐106b inhibitor/ mimics in rats

	Control	Vehicle	miR‐106b inhibitor	miR‐106b mimics
WBC (μL^−1^)	8736.4 ± 925.6	8901.7 ± 1078.4	8586.6 ± 876.5	8831.0 ± 943.6
Haemoglobin (g·L^−1^)	127.7 ± 11.2	130.0 ± 10.9	134.0 ± 18.0	135.3 ± 14.1
Platelets (×10^9^ L^−1^)	604.8 ± 72.0	579.7 ± 48.2	590.2 ± 40.6	551.4 ± 54.9
BUN (mmol·L^−1^)	5.2 ± 0.63	5.3 ± 0.97	5.1 ± 0.83	5.3 ± 1.0
Creatinine (μmol·L^−1^)	49.6 ± 5.11	47.5 ± 4.57	46.6 ± 5.09	45.5 ± 6.09
AST (IU·L^−1^)	138.0 ± 15.0	141.5 ± 20.5	149.1 ± 12.4	152.2 ± 22.0
ALT (IU·L^−1^)	45.1 ± 10.5	41.8 ± 11.1	47.1 ± 12.5	44.0 ± 10.5

Abbreviations: ALT, alanine aminotransferase; AST, aspirate aminotransferase; BUN, blood urea nitrogen; WBC, white blood cell.

## 4 DISCUSSION

MiR‐106b is located on the 13th intron of the mini‐chromosome maintenance protein 7 gene and belongs to the miR‐106b‐25 cluster, which has been reported to be an oncogene associated with certain cancers, such as colon cancer, oesophageal cancer and hepatocellular carcinoma.^31^, ^39^, ^40^ Previous research has shown that the inhibition of miR‐106b lightened hormone‐induced osteoporosis through the BMP2/SMAD signalling pathway.^18^ In addition, inhibiting the expression of miR‐106b has been demonstrated to reduce the degree of joint inflammation and joint bone destruction in mice with rheumatoid arthritis by inhibiting synovial inflammation, regulating RANKL/OPG signalling and reducing the number of mature osteoclasts.^19^ Therefore, our study further clarified the key role and potential mechanism of miR‐106b in Ti particle‐induced osteolysis by exploring the effect of miR‐106b in the treatment of PPO.

The maintenance and changes in bone tissue depend on the dynamic balance between bone formation by osteoblasts and bone resorption by osteoclasts.^41^ In PPO, bone regeneration was weakened, and bone resorption was strengthened.^26^, ^42^ Thus, suppressing the formation and function of osteoclasts and improving the osteogenic ability of osteoblasts are ideal methods to inhibit osteolysis in PPO. In this study, the vehicle and miR‐106b mimics‐treated groups showed a marked increase in TRAP‐positive areas around the Ti implants relative to the control group. However, after inhibition of miR‐106b, the area of TRAP‐positive staining was significantly reduced. Moreover, miR‐106b inhibition also relatively increased ALP‐positive multinucleated cells around the Ti implants. Our results demonstrated that the miR‐106b inhibitor decreased bone resorption and enhanced bone formation by regulating the balance between osteoblasts and osteoclasts and, as a result, was protective against bone loss.

In this study, we systematically investigated the effect of miR‐106b on PPO in rats. Our results showed that miR‐106b regulated the balance of osteoclasts and osteoblasts through the PTEN/Akt and NF‐κB signalling pathway, as well as by regulating macrophage polarization. Thus, it was possible to conclude that miR‐106b plays a crucial role in Ti particle‐induced osteolysis, and as such, the miR‐106b inhibitor is a potential option for preventing PPO.

Previous studies have shown that miR‐106b is involved in inflammatory responses,^19^ and we demonstrated here that the protective effect of miR‐106b in Ti particle‐induced osteolysis was related to macrophage polarization. As one of the most diverse cell types, macrophages can be activated in various ways by the tissue microenvironment and bioactive factors. M1‐polarized macrophages secrete a series of pro‐inflammatory factors that rising inflammatory responses. When M1 macrophage activation is dominated in the implant, inflammation exists, and the implant enters the stage of chronic deferment, which can lead to fibrous proliferation around the implant, blocked bone formation, enhanced bone resorption, and eventually, implant failure.^43^ In contrast, M2 macrophages secrete anti‐inflammatory factors and engulf necrotic tissue debris, thereby maintaining the stability of the tissue microenvironment. Therefore, macrophages play a key regulatory role in the inflammatory response in PPO. Our study showed that a large number of M1 macrophages were clustered in the fibrous capsule between the bone matrix and Ti rod, whereas miR‐106b inhibition significantly reduced the growth of Ti‐induced M1 polarization and increased the number of M2 macrophages. Accordingly, miR‐106b inhibition reduced the expression of pro‐inflammatory cytokines that contributed to increased osteoclastic bone erosion.^44^ Thus, we can infer that miR‐106b inhibition alleviated Ti‐induced bone destruction by regulating macrophage polarization and inhibiting a series of subsequent inflammatory responses.

This study also verified the underlying mechanism of miR‐106b in bone resorption. There is valid evidence that miR‐106b can regulate multiple target genes related to bone metabolism.^17^ PTEN, a well‐known tumour suppressor gene, is also involved in the regulation of bone formation and resorption and has been confirmed as a target of miR‐106b.^31^, ^32^, ^45^ The substrate of PTEN is a lipid produced by P13K, which is necessary for the activation of Akt (also known as protein kinase B (PKB)). PTEN regulates the activity of Akt by controlling the activation of PI(3,4,5)P3. PTEN dephosphorylates PIP3 to antagonize the activity of P13K, which inhibits the activation of Akt. It is through this mechanism that PTEN regulates cellular activities and participates in various biological processes, such as tumour biology and angiogenesis.

Several studies have been conducted to investigate the role of PTEN in bone metabolism. Blüml et al reported the effect of the PI3K/ PTEN axis on inflammatory bone destruction.^46^ They observed that PTEN deficiency increased the osteoclast development in vivo and in vitro by enhancing the expression of the RANKL‐induced osteoclast transcription factor, and in a human TNF‐transgenic arthritis model, there was increased local osteoclast activity in the joints. Hyun et al demonstrated that the loss and inhibition of PTEN phosphorylation promoted osteoclast formation by increasing the activity of Akt.^47^ Interestingly, miR‐214 was shown to promote osteoclastogenesis through the PTEN/PI3K/Akt pathway.^48^ These findings show that PTEN is a key regulator of Akt in osteoclast activation. Considering the pivotal role of the PI3K/Akt axis in osteoclastogenesis and osteoblast differentiation,^49^, ^50^ it is reasonable to speculate that miR‐106b is a target for the treatment and prevention of PPO by modulating the PTEN/PI3K/Akt pathways. Our results demonstrated that miR‐106b inhibition resulted in the inhibition of the PI3K/Akt pathway and RANKL activation.

In addition to the above presented information, previous studies have shown that NF‐kB plays an important role in inflammatory processes and its activation may be mediated by PI3K/AKT. Studies have shown that NF‐κB plays an important role in inflammatory processes.^51^ Moreover, NF‐κB has been found to be an important regulator of wear particles‐induced osteolysis and bone remodeling.^34^, ^38^, ^52^, ^53^ Some scholars have found that blocking NF‐κB activation inhibits inflammatory bone destruction.^22^ Notably, in this study, we found that inhibition of miR‐106b suppressed the phosphorylation of IκB‐α and p65, which resulted in the inhibition of NF‐κB signal activation in osteoclasts. These data suggested that the miR‐106b inhibitor suppressed inflammation‐induced bone destruction by inhibiting the NF‐κB signalling pathway.

In summary, in this study, we successfully established a rat implant wear debris model, and a miR‐106b inhibitor and miR‐106b mimics were used as interventions in this model. We subsequently found that an inhibitor of miR‐106b could alleviate wear particles‐induced osteolysis by promoting bone formation and inhibiting both osteoclast differentiation and macrophage‐mediated inflammation. In addition, we confirmed that miR‐106b inhibition prevented the expression of PTEN and blocked the PI3K/AKT and NF‐κB signalling pathways, which may be the mechanism that regulates the anti‐osteolytic effect of miR‐106b inhibition, as well as macrophage polarization. Our results suggest that miR‐106b may be a promising target for the treatment of PPO and aseptic loosening.

## CONFLICT OF INTEREST

The authors declare that they have no conflict of interest.

## AUTHOR CONTRIBUTIONS

BY, JB, JS, YX, QQ and DG designed the research study; BY, JB, JS, JS, XG, YL, GG and JL performed the experiments; BY, JB and DG analysed the data; BY, JB, JS, YX and DG wrote the manuscript; BY, JB, YT, HL and QQ revised the manuscript. All authors approved the final version to be published.

## Data Availability

The data used to support the findings of this study are available from the corresponding author upon reasonable request.
